# Ligand autoradiographical quantification of histamine H_3_ receptor in human dementia with Lewy bodies

**DOI:** 10.1016/j.phrs.2016.08.034

**Published:** 2016-11

**Authors:** Natasha L. Lethbridge, Paul L. Chazot

**Affiliations:** Department of Biosciences, University of Durham, Durham, UK

**Keywords:** DLB, Dementia with Lewy Bodies, AD, Alzheimer’s Disease, ADHD, attention deficit hyperactivity disorder, NFTs, neurofibrillary tangles, PM, post mortem delay, RαMHA, R-α-methylhistamine, MMSE, Mini Mental State Examination, UPDRS, Unified Parkinson Disease Rating Scale, MCID, Microcomputer Imaging Device, Human, DLB, Psychosis, Globus pallidus, Histamine, H_3_R

## Abstract

Dementia with Lewy bodies (DLB) is a serious age-dependent human neurodegenerative disease, with multiple debilitating symptoms, including dementia, psychosis and significant motor deficits, but with little or no effective treatments. This comparative ligand autoradiographical study has quantified histamine H_3_ receptors (H_3_R) in a series of major cortical and basal ganglia structures in human DLB and Alzheimer’s (AD) post-mortem cases using the highly selective radioligand, [^3^H] GSK189254.

In the main, the levels of H_3_ receptor were largely preserved in DLB cases when compared with aged-matched controls. However, we provide new evidence showing variable levels in the globus pallidus, and, moreover, raised levels of Pallidum H_3_ correlated with positive psychotic symptoms, in particular delusions and visual hallucinations, but not symptoms associated with depression. Furthermore, no correlation was detected for H_3_ receptor levels to MMSE or IUPRS symptom severity.

This study suggests that H_3_R antagonists have scope for treating the psychotic symptomologies in DLB and other human brain disorders.

## Graphical abstract

**Figure:**
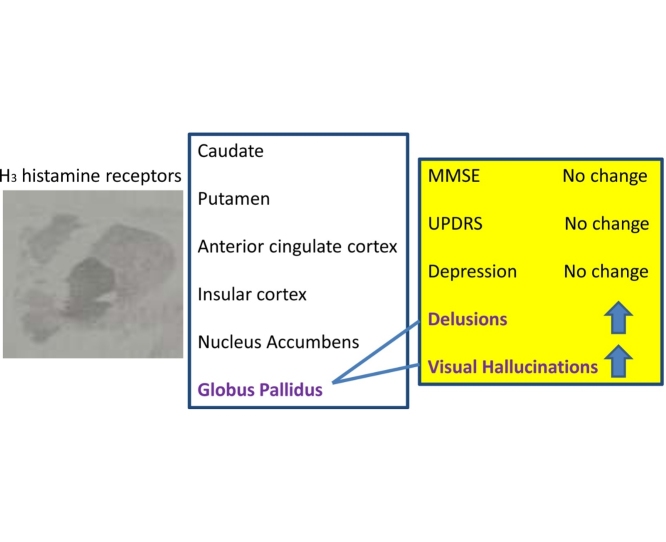

## 1 Introduction

Dementia with Lewy bodies (DLB) is the second most prevalent human dementia. This is a seriously debilitating human disease, with multiple prevalent symptoms, including dementia, psychosis (hallucinations and fluctuating consciousness) and significant motor deficits, but with little or no effective treatments [1]. The histaminergic system plays an important role in central nervous system regulation and behaviour through its role as an autoreceptor, regulating the synthesis and release of histamine and as a heteroreceptor, negatively regulating the release of a variety of other key neurotransmitters including acetylcholine, dopamine, glutamate and gamma-aminobutyric acid [2], [3], [4]; reviewed in [5]. Given its widespread distribution and influence upon multiple neurotransmitter systems, H_3_ antagonists are promising clinical candidates for the treatment of age-related dementias, such as DLB [6], [7], [8].

There are indications that histamine deficits are present in dementias, such as Alzheimer’s Disease (AD), however it is unknown whether these are specific to certain brain regions, changes in histamine receptor numbers, or are specific for AD amongst other neurodegenerative disorders. The importance of the histaminergic system in AD is difficult to assess due to a number of conflicting reports. For example, histamine levels in AD brains have been reported to be increased in temporal and frontal cortex, basal ganglia and hippocampus [9]. However, other studies have shown decreases in histamine content in the hypothalamus, hippocampus and temporal cortex [10], [11]. Histaminergic cell bodies are also located in the TMN, where neurofibrillary tangles (NFTs) are also found. NFTs are particularly concentrated in the region containing histaminergic perikarya compared with surrounding areas [12], [13] and together with cholinergic basal forebrain nuclei, the TMN has been described as an early affected subcortical nucleus for the presence of NFT [14]. The number of histaminergic cell bodies in the TMN was shown to be similar to that of normal brains [12]. In contrast, another group showed a significant reduction in large-sized histamine containing neurons in the TMN where numerous NFTs were found, indicative of a central histaminergic dysfunction [13]. Histamine decarboxylase (HDC) activity, also a common marker of the histaminergic system, has been shown to be decreased in AD compared with elderly controls [15]. Whilst there are conflicting data about the histamine content in the brain of AD patients, one recent study using a highly selective H_3_R ligand had shown the level of H_3_R expression to be unaltered in the late stages of human AD compared to age matched controls, as well as in TASTPM mice (a mouse model of AD) compared with wild type mice [6], [16].

Understanding the molecular structure of the H_3_R has increased considerably and a number of H_3_R antagonists have been identified and a few (pitolisant and GSK189254) have entered advanced clinical development focusing on narcolepsy, cognitive and psychotic disorders [8], [18], [19]. The histaminergic system innervates several structures that are known to be involved in cognition such as the basal forebrain, cerebral cortex, cingulate cortex, amygdala and thalamus [20]. High levels of H_3_R have been shown to be expressed in the cerebral cortex [21], which is densely innervated by cholinergic neurons. In neuropsychiatric disorders such as AD, attention deficit hyperactivity disorder (ADHD) and schizophrenia, cognitive deficits play a major role in the disease [22]. Increased brain histamine is also positively correlated with age and may play a role in decreasing acetylcholine uptake [23]. It is thought that H_3_R antagonists may be able to prevent the reduction in acetylcholine through its heteroreceptor characteristic [24], [25], [26]. H_3_Rs are also highly expressed in the basal ganglia in both rodent and human brains [27], [28], [29].

Ligand autoradiography is a very useful technique to define the topology and quantify receptors in post-mortem brain slices. GSK189254 is derived from a novel benzazepine series of H_3_R antagonists [6] that are structurally distinct from other recently described non-imidazole H_3_R antagonists. GSK18925 has been shown to significantly improve performance of rats in diverse cognition paradigms, including passive avoidance, water maze, object recognition and attentional set shift [4], [5]. The data thus far for H_3_R antagonists point to a possible therapeutic potential for diseases where cognitive deficits are already present such as AD and other dementias, including DLB. These complex brain diseases also display multiple symptoms in addition to dementia which may be targeted through the histaminergic system. In this present study, [^3^H] GSK189254 was utilised to quantify levels of cortical and basal ganglia H_3_Rs in normal human aged post-mortem brains, and in a series of DLB and AD cases (the latter for comparative purposes) with detailed connected clinical information.

## 2 Materials & methods

### 2.1 Determining the working concentration of [^3^H] GSK189254 for autoradiography

Saturation binding assays using [^3^H] GSK189254 were performed essentially as described previously [6], in 50 mM Tris-HCl, pH 7.7 containing 5 mM EDTA and a concentration range of 0.01–8 nM for radioligand. Non-specific binding was determined using 1 μM R-α-methylhistamine (RαMeH). The assay was terminated by rapid filtration through a Whatman GF/B filters pre-soaked in 10 mM sodium phosphate dibasic pH 7.4, which were washed (3 × 3 ml) using iced cold 10 mM sodium phosphate dibasic pH 7.4, using a Brandell 24-place cell harvester.

[^3^H] GSK189254 bound selectively to the hH_3_R vs hH_4_R, and the two major hH_3_R isoforms, namely hH_3_ 445 and hH_3_ 365, transiently expressed in HEK293 cells [6], displayed very similar K_D_ values of 0.16 ± 0.04 and 0.24 ± 0.07 nM, respectively (Supplementary Fig. 1). The concentration of radioligand used was, therefore, selected as approximately 2× mean K_D_ to ensure that each autoradiography run detected at least 65% of available receptor binding sites.

### 2.2 Human case details and diagnostic criteria

All human brain tissue were obtained from Newcastle Brain Tissue Resource Bank LREC (Newcastle and Tyneside) with full ethical approval (2002/295). Frozen tissue was collected at autopsy and 1 cm coronal slices from the left hemisphere were snap frozen in liquid Arcton (ICI) and stored at −70 °C. The sections were then stored at −80 °C. Prior to sectioning, tissue slices were warmed to 15 °C and blocks containing the striatum were sub-dissected and mounted onto cryostat chucks with 8% carboxymethylcellulose. Coronal sections were cryostat sectioned at a thickness of 20 μm using a Brights OTF cryostat onto Vectabond-coated glass slides, air dried for 1–2 h and stored at −80 °C prior to receptor autoradiography. The right hemisphere was used for histopathological examination, following formalin fixation and paraffin embedding. Cortical and hippocampal neurofibrillary tangles were demonstrated using a modification of Palmgren’s silver technique [30] and the von Braunmühl silver impregnation technique [31] was used to identify senile plaques in 25 μm thick frozen sections cut from tissue blocks adjacent to those taken for paraffin processing. Counts of NFTs and neuritic plaque number were made from fields across the entire cortical ribbon, as described in [32]. Lewy-bodies in the substantia nigra were visualised by the use of haematoxylin and eosin staining, cortical Lewy-bodies and dystrophic neuritis were detected using ubiquitin immunohistochemistry on 5 μm thick paraffin embedded sections. Neurones in the substantia nigra were quantified following cresyl fast violet staining of 20 μm thick paraffin sections.

Control cases had no history of psychiatric or neurological disorder and had no neuropathological indications of Lewy-body disease (DLB) or any other neurological disorder. DLB cases were clinically diagnosed by the presence of a progressive cognitive impairment seen in conjunction with at least two of the following symptoms: recurrent visual hallucinations; fluctuating cognition with pronounced variations in attention and alertness; spontaneous motor features of parkinsonism [33]. DLB cases were distinguished from AD by the presence of brain stem and cortical Lewy-bodies, Lewy neurites in the CA2/3 and endplate segments of the hippocampus [33], and by lower or moderate Alzheimer-type pathology with fewer NFT than found in AD.

### 2.3 Human cases used

The 43 cases selected for this study were cut at the level of the striatum (caudate nucleus and putamen) corresponding to coronal brain levels 9–15 using the Coronal Map of Brodmann Areas in the human Brain [34]. Of the 43 cases, 12 were control cases, 16 DLB cases and 15 AD cases (Table 1). For each case 5 replicates were used to measure 3 total and 2 non-specific radioligand binding.

Summary of the 43 human cases chosen for the study. PM delay = post mortem delay, that is, time between death and freezing of the tissue to allow for post-mortem examination.

No significant differences were seen with age or PM delay in these cases (p > 0.05). No gross significant differences were seen between the male and female cases in respective groups (p > 0.05) (not shown).

### 2.4 In vitro autoradiography of human brain tissue using [^3^H] GSK189254

The autoradiography method used was essentially as described previously [6]. In brief, human brain sections were left to equilibrate to room temperature for 1 h before the protocol commenced. Human sections were incubated in (50 mM Tris, 5 mM EDTA pH 7.7) containing 2 × K_D_ (approximately 0.5 nM) [^3^H] GSK189254 (specific activity = 81Ci/mmol, stored at −20 °C, gift from Dr Medhurst, GSK, Harlow, UK) for 1 h at RT, until equilibrium is reached. Non-specific binding was defined using 10 μM unlabelled RαMHA. The reaction was terminated by five 3 min washes in 50 mM Tris, 5 mM MgCl pH 7.7, at 4 °C and a final wash in dH_2_O at 4 °C. Sections were left to dry in a stream of cold air for 1–2 h. The sections were then transferred to X-ray cassettes, each including tritium autoradiographical microscale as calibration standards, and exposed against tritium-sensitive hyperfilm for 6 weeks at 4 °C. The exposed films were then developed in D-19 developer (Kodak, UK) for 5 min at RT, fixed for 6 min in Unifix (Kodak, UK), washed under running water for 20 min and air-dried.

### 2.5 Image analysis

The resulting brain images on the film were captured using a Dage 72 MTI CCD72S video camera and were quantitatively analysed by computer-assisted densitometry using Microcomputer Imaging Device (MCID Elite) version 7.0 software from imaging research Inc., Ontario, Canada. The radioactive Tritium standards were used to calculate a standard curve for each autoradiogram, which allowed the conversion from optical density values to units of concentrations for each brain region analysed. Non-specific binding tissue sections were present on the same film as each of the corresponding total binding tissue sections for the same case. Specific binding was determined by subtracting mean non-specific binding from mean total binding. Brain structures were identified by reference to the atlas of the Human Brain [34] and the mean and standard deviations for each brain structure in each section were calculated. Inter-assay variability was reduced by using ligand concentrations that were at least twice the ligand affinity, using ligand from the same batch for each autoradiographical run, and by standardising each film using calibration microscales. All sections were then re-analysed and results confirmed by digital autoradiography using a Beta-Imager 2000 instrument (Biospace, Paris, France), radioactivity was measured by counting the number of β particles from delineated areas and the results are expressed as mean specific binding counts per minute per square millimetre (cpm/mm^2^; *n* = 12–16 cases per group).

### 2.6 Symptom analysis

#### 2.6.1 The mini mental state examination (MMSE)

The MMSE, validated and widely used since its creation in 1975, is an effective tool for assessing cognitive mental status. The MMSE is used to detect cognitive impairment and monitor response to treatment. It is an eleven question test covering five areas of cognitive function: orientation, attention/calculation, recall and language, and the ability to follow simple verbal and written commands [35]. A score of 23 or below, from a possible 30 is indicative of cognitive impairment. The test is effective but does have limitations, for example, patients who are hearing and visually impaired or who have low English literacy, or with communication disorders may perform poorly even when cognitively intact [35]. The test provides a total score that places the individual on a scale of cognitive function. The values used in this were those taken at the last assessment before death of the patient.

#### 2.6.2 Unified Parkinson disease rating scale (UPDRS)

The UPDRS is a rating tool to follow the longitudinal course of PD. It is made up of the 1) Mentation, Behaviour and Mood, 2) Activities of Daily Living (ADL) and 3) Motor sections. These are evaluated by interview. Some sections require multiple grades assigned to each extremity. A total of 199 points are possible, where 199 represent the worst (total) disability, and 0 represents no disability [36]. The values used in this study were those taken at the last assessment before death of the patient.

Estimated lines of best fit for MMSE and UPDRS correlations were produced using GraphPad Prism and are represented on each graph, indicating any changes in binding levels in each tissue with increasing clinical score. The significance of the regression was determined from the generated p value, where p ≤ 0.05 was considered to show a significant linear relationship between clinical score and binding level.

#### 2.6.3 Other symptom analysis

Data relating to depression, delusions, dementia and visual hallucinations experienced by each subject in life were also studied. The severity of the symptoms experienced were measured on the following scale, 0 = none, 1 = mild, 2 = severe, and are indicative of the last assessment before death of the subject. In each case and in each tissue investigated, attempts were made to correlate the specific binding levels of [^3^H] GSK189254 data with a range of relevant clinical data scores. The depression, delusion and visual hallucination scores were displayed: 0 no symptoms and 1+ showing symptoms, giving the mean score ± SD against binding levels in cpm/mm^2^.

### 2.7 Statistical analysis

Statistical analysis performed involved correlation analysis and students unpaired *t*-test, to analyse individual regions of the brain. Graphs and one-way ANOVA with appropriate post-hoc test were constructed using GraphPad Prism version 4. Statistical significance was set at p < 0.05.

## 3 Results

### 3.1 Human H_3_R pharmacology of [^3^H] GSK189254

The selectivity of [^3^H] GSK189254 for the human H_3_R 445 in comparison to the closely related human H_4_R 390 subtype was investigated. No significant binding was observed for the hH_4_ receptor (Supplementary Fig. 1) Moreover, very similar Kd values (ca. 0.3 nM) for [^3^H] GSK189254 were observed with two of the most common, in cortical-striatal regions, human H_3_R isoforms, H_3_R 445 and H_3_R 365 expressed alone or in combination in HEK293 cells (Supplementary Fig. 1). A representative digital photographic examplar of specific [^3^H] GSK189254 binding shows the high levels of specific binding in the human brain slice. Very low non-specific binding (<5%) was achieved with the methodology utilised in this study. High binding levels were detected in various cortical (insular, anterior cingulate) and striatal (caudate, putamen, globus pallidus, nucleus accumbens) regions (Supplementary Fig. 1) all relevant to symptomology of DLB.

### 3.2 Age-dependence of [^3^H] GSK189254 binding in control and dementias

**Fig. 1:**
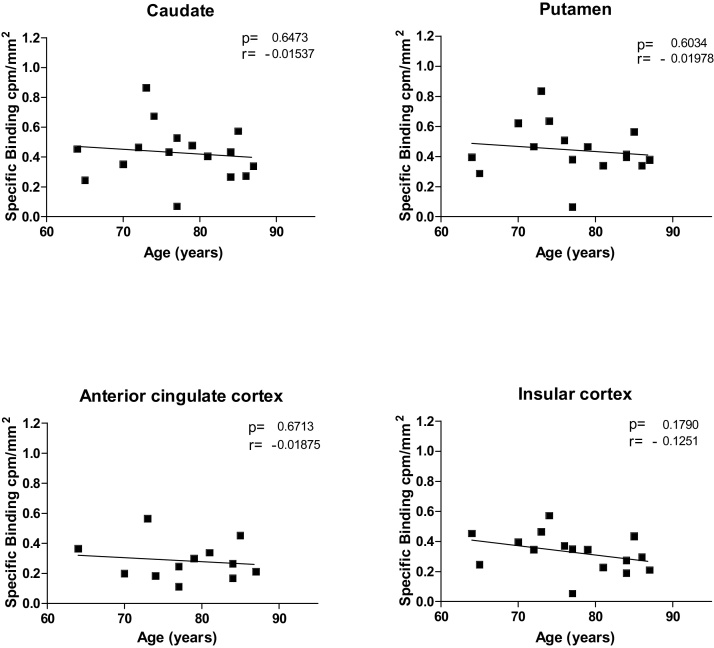
Age-dependent specific binding of [^3^H] GSK189254 in DLB cases (n = 16) in caudate, putamen, anterior cingulate cortex, insular cortex, nucleus accumbens and globus pallidus. No significant change in [^3^H] GSK189254 binding levels was see with the brain structures investigated (n = number of individual patient cases).

DLB cases were first examined for [^3^H] GSK189254 binding levels in the various cortical and striatal brain regions spanning an age range of 60–80 years. There were no significant age-dependent changes in all brain regions analysed although individual variation was clear (p ≥ 0.1 in all areas) (Fig. 1). A similar lack of change was observed in both control and AD cases over the age-range explored (not shown).

### 3.3 [^3^H] GSK189254 binding levels in control and dementia cases

**Fig. 2:**
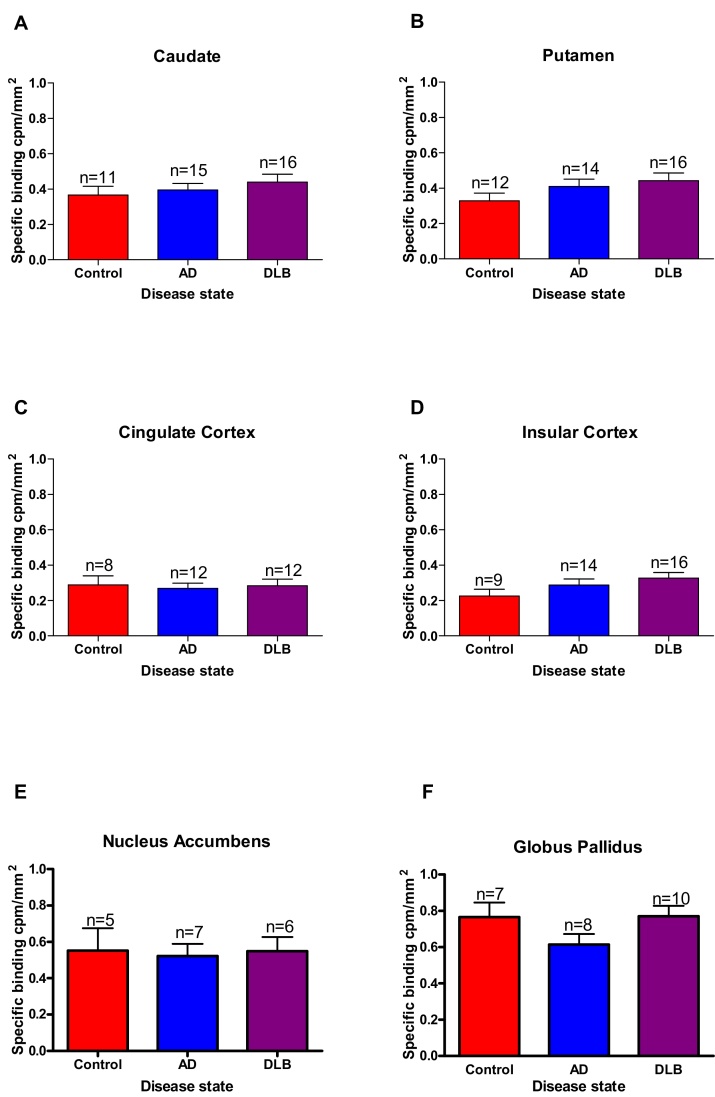
[^3^H] GSK189254 specific binding (cpm/mm^2^) densities (mean ± SEM for n individual patient cases) for pooled Control, DLB and AD cases for (A) Caudate, (B) Putamen, (C) Cingulate cortex, (D) Insular cortex, (E) external Globus Pallidus, (F) internal Globus Pallidus. No significant differences in binding levels was observed in the brain structures investigated.

As there were no clear changes in [^3^H] GSK189254 binding levels across the age-range, the data sets were pooled and a comparison made between controls and the two dementias. No significant differences were observed in the mean binding densities of [^3^H] GSK189254 binding in all brain regions analysed (Fig. 2). The data were further analysed for gender differences (**not shown**), similar levels of binding was seen in both female and male cohorts in all brain regions, apart from minor changes in the globus pallidus, indicating little or no evidence for gender bias.

### 3.4 Correlation of [^3^H] GSK189254 binding levels to cognitive and motor deficits

**Fig. 3:**
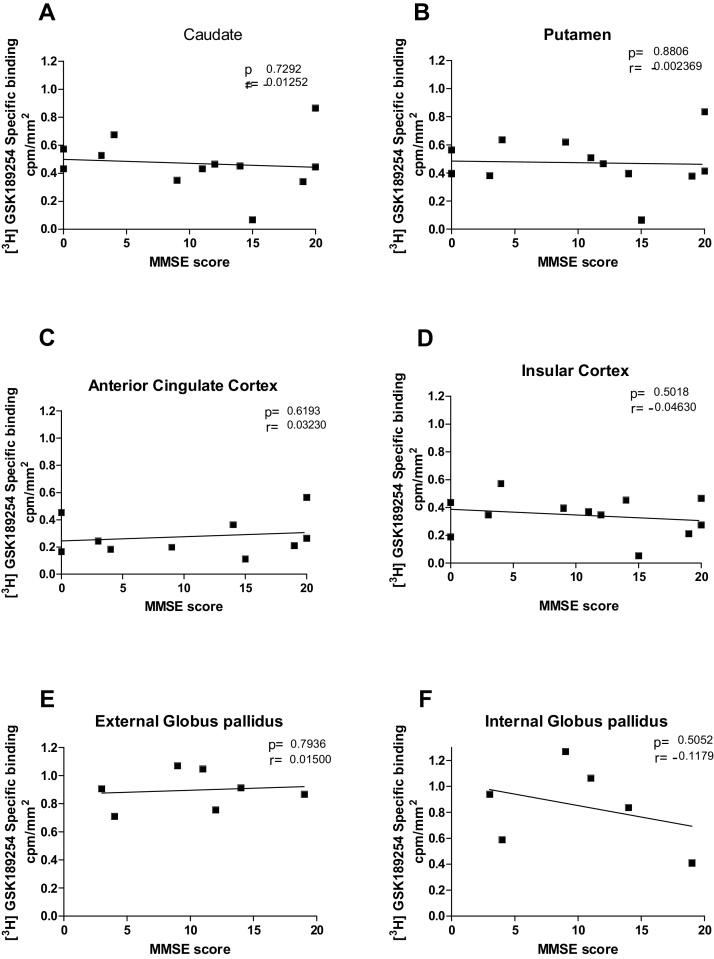
MMSE Scale against specific binding cpm/mm^2^ of [^3^H] GSK189254 in DLB cases in (A) Caudate, (B) Putamen, (C) Cingulate cortex, (D) Insular cortex, (E) External globus pallidus, (F) Internal globus pallidus. No significant relationship was see with the brain structures investigated (each point is an individual DLB patient case).

**Fig. 4:**
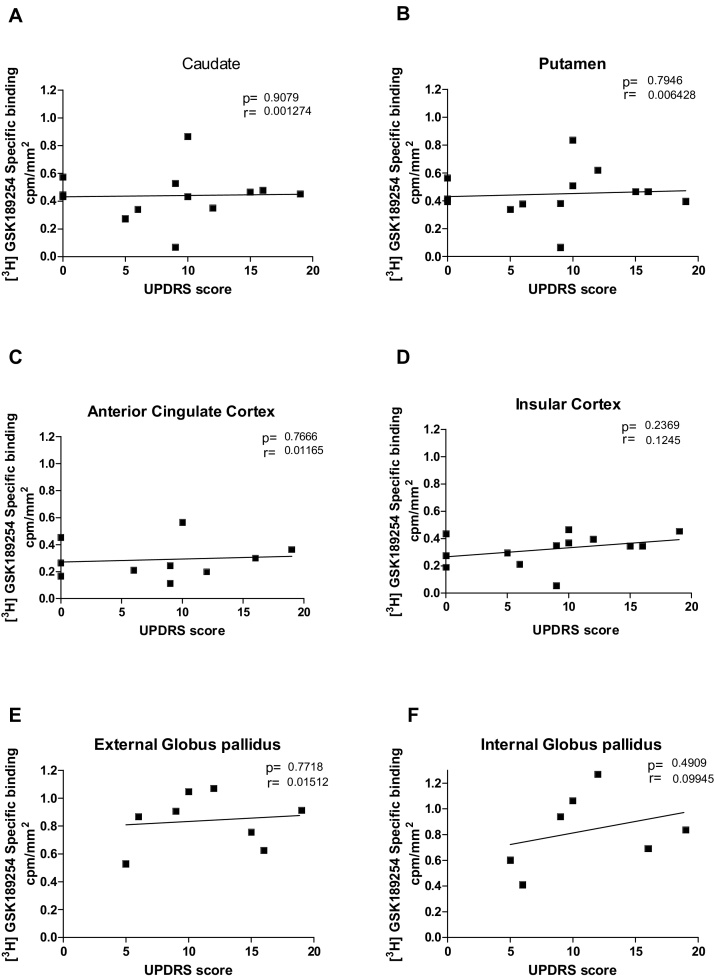
Unified Parkinson Disease Rating Scale against specific binding cpm/mm^2^ of [^3^H] GSK189254 in DLB cases in (A) Caudate, (B) Putamen, (C) Cingulate cortex, (D) Insular cortex, (E) External globus pallidus, (F) Internal globus pallidus. Each point is an individual DLB patient case.

The clinical data corresponding to DLB and AD cases summarised in Table 1 were further analysed to determine if there were any correlation between [^3^H]GSK189254 binding and MMSE (mini mental state examination) (Fig. 3) and UPDRS scores (Unified Parkinson disease rating scale) (Fig. 4). There was no significant correlation in the binding densities of [^3^H] GSK189254 with MMSE score (p ≥ 0.5) in all areas in DLB cases. There was also no significant correlation in the binding densities of [^3^H] GSK189254 with MMSE score in AD cases analysed in parallel (p ≥ 0.2 in all areas) (Supplementary Fig. 2).

Moreover, there were also no significant differences in the binding densities of [^3^H] GSK189254 with increased UPDRS score in DLB (Fig. 4) and AD cases (Supplementary Fig. 3).

### 3.5 Correlation of [^3^H] GSK189254 binding levels to affective and psychotic deficits

**Fig. 5:**
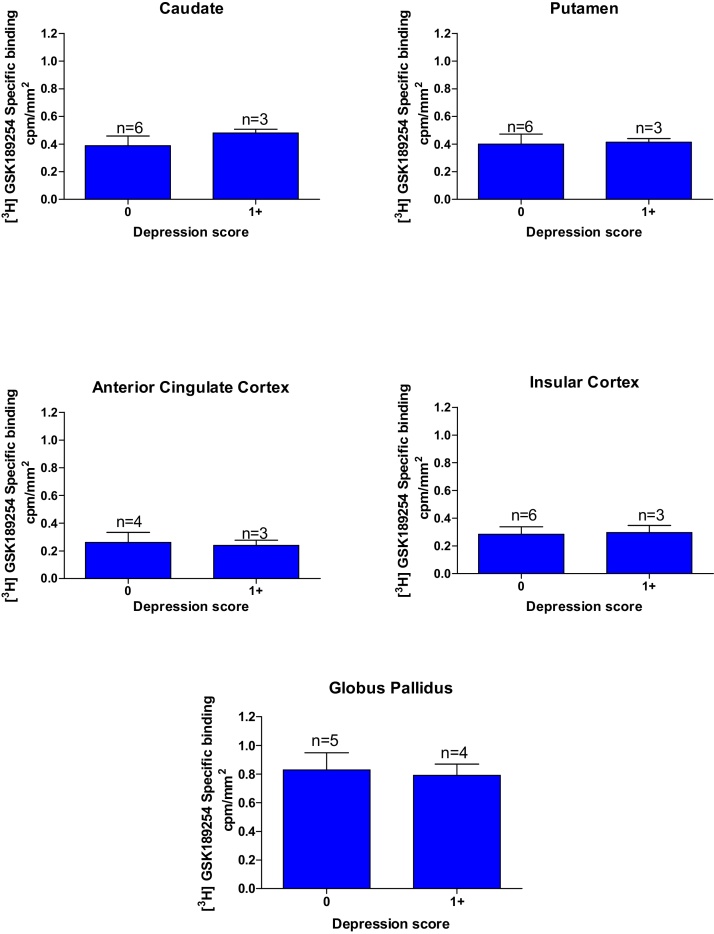
Correlation of Depression score against specific binding cpm/mm^2^ of [^3^H] GSK189254 in DLB cases in (G) Nucleus Accumbens, (H) combined Globus Pallidus. No significant correlation was observed between [^3^H] GSK189254 binding levels and depression scores in all brain structures investigated. N = number of individual Disease cases.

In many cases investigated, clinical information relating to depression and psychosis symptoms were recorded. There were no significant differences between the H_3_R binding densities in DLB (Fig. 5) and AD cases (Supplementary Fig. 3). with and without depression in all brain structures investigated.

**Fig. 6:**
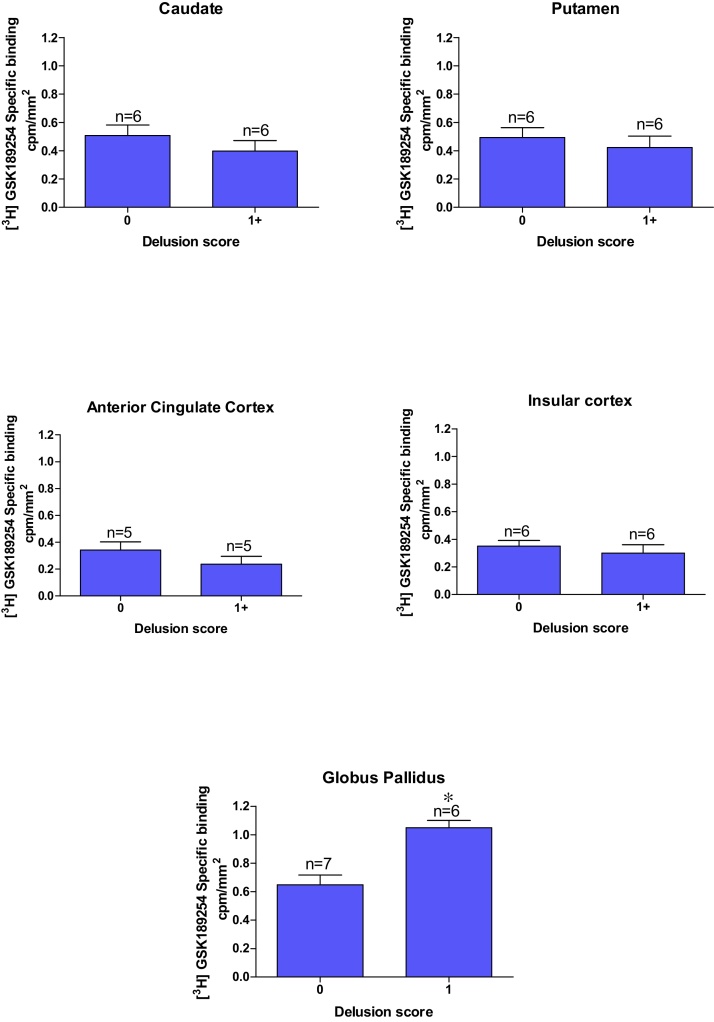
Correlation of delusion score against specific binding cpm/mm^2^ of [^3^H] GSK189254 in DLB cases in (G) Nucleus Accumbens, (H) combined Globus Pallidus. A significant elevation of [^3^H] GSK189254 binding sites in the globus pallidus was observed in DLB cases with severe delusion compared to cases lacking delusions (p < 0.05). No significant relationship was oberved with the other brain structures investigated. (n = number of individual patient cases).

Similarly, there were no significant differences between the H_3_R binding densities in DLB cases with and without severe delusions, except in the globus pallidus where a significant increase in H_3_R binding was observed in cases with severe delusions (p < 0.01) (Fig. 6).

There were no significant differences between the H_3_R binding densities and severity of visual hallucinations, although there is an increased H_3_R binding in the globus pallidus associated with severe visual hallucinations.

**Fig. 7:**
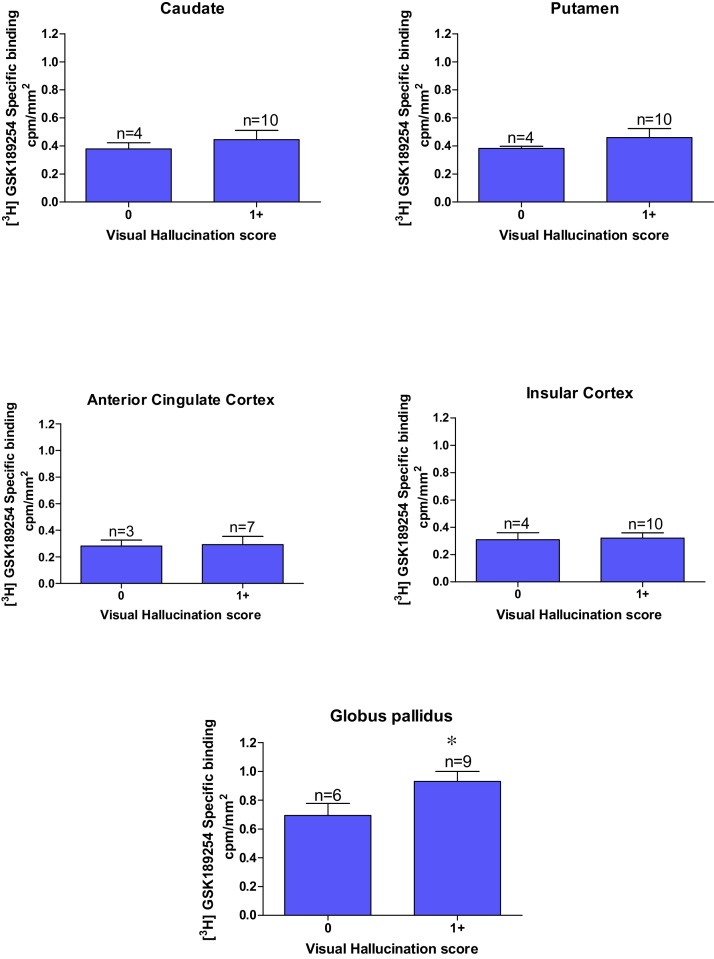
Visual hallucination score against specific binding cpm/mm^2^ of [^3^H] GSK189254 in DLB cases in (G) Nucleus Accumbens, (H) combined Globus Pallidus. A significant elevation of [^3^H] GSK189254 in the globus pallidus was observed in DLB cases with visual hallucinations (p < 0.05). No significant relationship was see with the other brain structures investigated (n = number of individual patient cases).

Overall H_3_R binding in both AD and DLB cases does not show any correlation with MMSE, UPDRS, and depression symptoms in cortical or striatal structures in the human CNS. In contrast, increased H_3_R binding positively correlated with increased severity of psychotic symptoms (delusions and visual hallucinations) in the globus pallidus in both DLB Fig. 6, Fig. 7 and AD (Supplementary Fig. 4 and 5) cases.

## 4 Discussion and conclusion

The high affinity and selective H_3_R antagonist/inverse agonist [^3^H] GSK189254 provides an ideal tool to visualise and allow quantification of the human histamine H_3_R. The ligand displays a high affinity for two of the most common human H_3_R isoforms, and also very low non-specific binding properties, which makes it an ideal ligand autoradiographical tool and a vast improvement on previously utilised radioligands (e.g. RαMethylhistamine and clobenpropit [37]). A range of brain structures implicated in the characteristic symptoms of DLB and AD were investigated. The striatum has a well-known role in planning and modulation of movement pathways, but is also involved in a variety of other cognitive processes involving executive function. The cerebral cortex is involved in many complex brain functions including memory processing, attention, perceptual awareness, language and consciousness. More specifically, the anterior cingulate cortex and globus pallidus are thought to be major neuroanatomical interface between emotion and cognition, and the insular cortex is believed to process convergent information to produce an emotionally relevant context for sensory experience. The main focus of this present study was to determine any changes in the H_3_R in relation to age and gender in control, DLB and AD cases, and relationship to specific symptoms displayed by the individuals. Several lines of evidence suggest that manipulation of the histamine system may alleviate some of the clinical symptoms of AD and DLB. H_3_R blockade with antagonist/inverse agonists results in the up-regulation of several neurotransmitters which have been shown to have positive affects upon cognitive deficits in several animal models of dementia (reviewed in [8] and [11]).

Previous ligand autoradiography studies using less selective H_3_R radioligands have reported high H_3_R densities in the internal and external segments of the globus pallidus, caudate, putamen and nucleus accumbens with moderate levels in the anterior cingulate and insular cortices [38], [39] which concurs well with the present study. Furthermore, in this present study, no significant differences were observed with age although this was only over the restricted 60–80 age range; changes prior to 60 years of age may have occurred and require further investigation. Using [^3^H] clobenpropit it was also reported that no significant age-related changes in H_3_R expression in the basal ganglia occurred in normal ageing, nor did receptor density differ significantly between male and female cases [37]. Therefore, H_3_R levels does not appear to be grossly altered in the latter stages of normal aging.

H_3_R binding levels were next determined in DLB to establish whether disease state alters receptor levels. H_3_R binding densities in both cortical and striatal regions in DLB human cases showed no significant differences in ligand binding with age, which supported previously published data suggesting that the H_3_R is preserved in two common age-related dementias, namely AD and vascular dementias, in other cortical and limbic regions [40]. This was also confirmed in this present study with different AD cases and different cortical brain structures. Overall, these data suggest that there is no gross decline in H_3_R population between control and disease cases, providing further evidence for H_3_R preservation across a range of neurodegenerative diseases. Preclinical trials have already alluded to the prospect of H_3_R antagonists as a treatment for cognitive impairment. We provide further evidence showing preservation of H_3_Rs in many cortical and striatal brain regions in AD but also in DLB, promoting the H_3_R as a viable general target in treating a range of human dementias. This has yet to be realised in the clinic.

The data set produced was further interrogated with respect to selective symptoms present in the dementia cohorts prior to death and relationship to H_3_R expression. The H_3_R binding levels were correlated with symptom severity scores from various validated clinical tests. There was no correlation between H_3_R binding levels and MMSE or UPDRS scores in both DLB and AD cases, indicating that the H_3_R expression levels in the brain structures investigated do not influence the severity of cognitive and mobility impairment, respectively. The latter is in contrast with reported higher H_3_R binding levels observed in the motor loop structures, substantia nigra and ventral striatum in PD animal studies (e.g. [41]). These translational discrepancies highlight the importance of promoting more human postmortem and live imaging brain studies. There was a modest overall increase in H_3_R binding sites with decrease in MMSE score indicative of cognitive function. The increase in H_3_R binding maybe acting as a compensatory mechanism to counteract changes seen elsewhere in the histaminergic system in severe AD and DLB such as a decrease in frontal cortex H_1_R in AD [42], and reduced H_2_R expression in the hippocampus in both AD and DLB cases [35]. The functional consequence of increased H_3_R density could be a further decrease in cognitive neurotransmitters and hence further exacerbation of cognitive deficits, and so would not be a positive compensatory effect. Alternatively, the increase in H_3_R binding in brains of individuals with more severe dementia could be simply related to loss of cholinergic neurons. Loss of cholinergic neurons in the basal forebrain is one of the most prominent and consistent events occurring in AD [43]. These data support previous literature indicating that higher H_3_R binding correlated with more severe dementia (MMSE) in AD [16], but this was more pronounced in the pre-frontal cortex.

Now to consider other symptoms present in many of the cases studied. There was no correlation between H_3_R binding and severity of depression in DLB and AD cases, suggesting that the H_3_R does not play a major role in depression symptoms associated with AD and DLB. This is consistent with recent studies in depressed and bipolar patients [44], [45]. This lack of correlation held for most brains studied herein in terms of the psychotic symptoms. However, an interesting exception was the globus pallidus, where H_3_R binding levels positively correlated with presence of significant psychotic symptoms, particularly levels of delusion and, to a lesser extent visual hallucinations, in both DLB and AD cases. DLB cases with moderate to high delusion and visual hallucination scores displayed approximately 40% and 22% higher globus pallidus H_3_R binding densities, respectively, in comparison to cases lacking such psychotic symptoms. A similar trend was present in AD cases with moderate to high delusion and visual hallucination scores displayed approximately 37% and 14% higher H_3_R binding densities, respectively in comparison to cases lacking such psychotic symptoms. It has been previously reported that the globus pallidus is spared of pathology in Lewy body diseases, DLB and PDD [46]. However, the volume of the human globus pallidus has also been positively correlated with the severity of global psychotic symptoms, as measured by both the Scale for the Assessment of Negative Symptoms and Positive Symptoms [47], which may account for this apparent increase in the H_3_R. This finding was more profound in the DLB cases than AD cases and this is to be expected since DLB cases have generally more pronounced psychotic symptomology than AD cases. H_3_R expression has been shown to be altered in patients with Schizophrenia and is thought to be involved in the underlying neuropathology [48]. The study showed significantly higher histamine H_3_R radioligand binding sites in the prefrontal cortex of the schizophrenic group and bipolar subjects with psychotic symptoms, and higher H_3_R binding correlated with psychotic symptoms, as seen in this present study [48]. H_3_Rs in the human prefrontal cortex is thought to be involved in the modulation of cognition and emotional behaviours, and this is supported by findings in animals that H_3_R antagonists enhance prepulse inhibition and cognitive performance [49], [50], [51]. Early promise with pitolisant, a H_3_R antagonist/inverse agonist for the psychotic symptoms in schizophrenic patients [18], [19], has not been confirmed with another H_3_R antagonist, ABT-288 [17], with a distinct pharmacokinetic profile. Such studies are still lacking, however, in Lewy body dementia patients, DLB and PDD. The main limitation common to this type of study lies in the relatively small number of cases investigated. The quality of the case tissue and respective clinical information from a leading DLB brain bank centre is a strength of this study, but naturally, further studies are required to confirm these interesting findings utilizing cases from other international brain banks. Furthermore, future studies are also required to probe other key brain structures implicated in psychotic symptoms in DLB and PDD cases.

In conclusion, the key novel findings were the general preservation or elevated levels of the H_3_R in both normal ageing humans and in the two major human dementia disorders in a variety of cortical and striatal brain structures. This study reports, for the first time, the globus pallidus as a potential new player in the neuropathology of Dementias, particularly those with psychotic symptomologies such as DLB, and as a potentially new target for histaminergic clinical manipulation.

## Conflict of interest

None.

## Figures and Tables

**Table 1:** Summary of 43 human cases.

	n =			Age (years)			PM Delay (hours)		
	Total	Females	Males	Range	Mean	SD	Range	Mean	SD
Control	12	7	5	70–91	80.92	6.97	10–96	42	22.44
DLB	16	8	8	64–87	77.13	7.19	4–60	31.56	18.18
AD	15	9	6	74–91	83.27	4.53	4–82	33.40	21.69

Summary of the 43 human cases chosen for the study. PM delay = post mortem delay, that is, time between death and freezing of the tissue to allow for post-mortem examination.
