# Supplementary material for: Ligand autoradiographical quantification of histamine H3 receptor in human dementia with Lewy bodies
Source: Pharmacol Res. 2016 Nov;113(Pt A):245–56. doi: 10.1016/j.phrs.2016.08.034 (PMC5113906; doi:10.1016/j.phrs.2016.08.034)
Supplement: Supplementary file 1 [file mmc1.pdf]

A

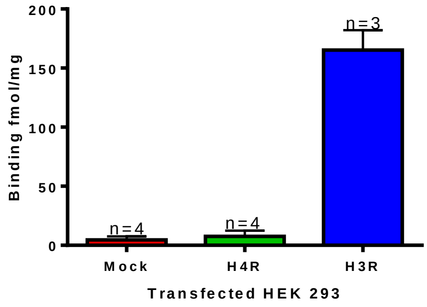

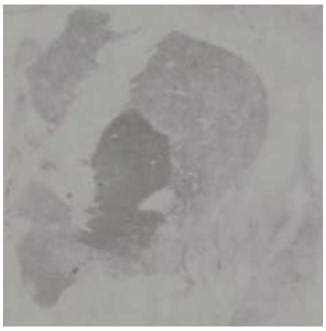

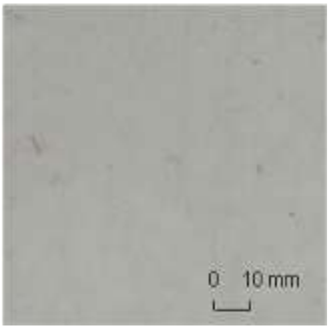

Total binding Non-specific binding

Supplementary Figure 1

A. Selective binding to hH_3R. [^{3}H] GSK189254 (1 nM) binding to mock transfected HEK293 cells, hH_3R and hH_4R respectively. Non-specific binding was defined using 10µM R-α-methylhistamine

B. Representative autoradiograms of human brain slices (87 years, female). (A) Total binding [^{3}H] GSK189254 (0.5 nM), (B) Non-specific binding [^{3}H] GSK189254. Non-specific binding defined using 10µM R-α-methylhistamine.

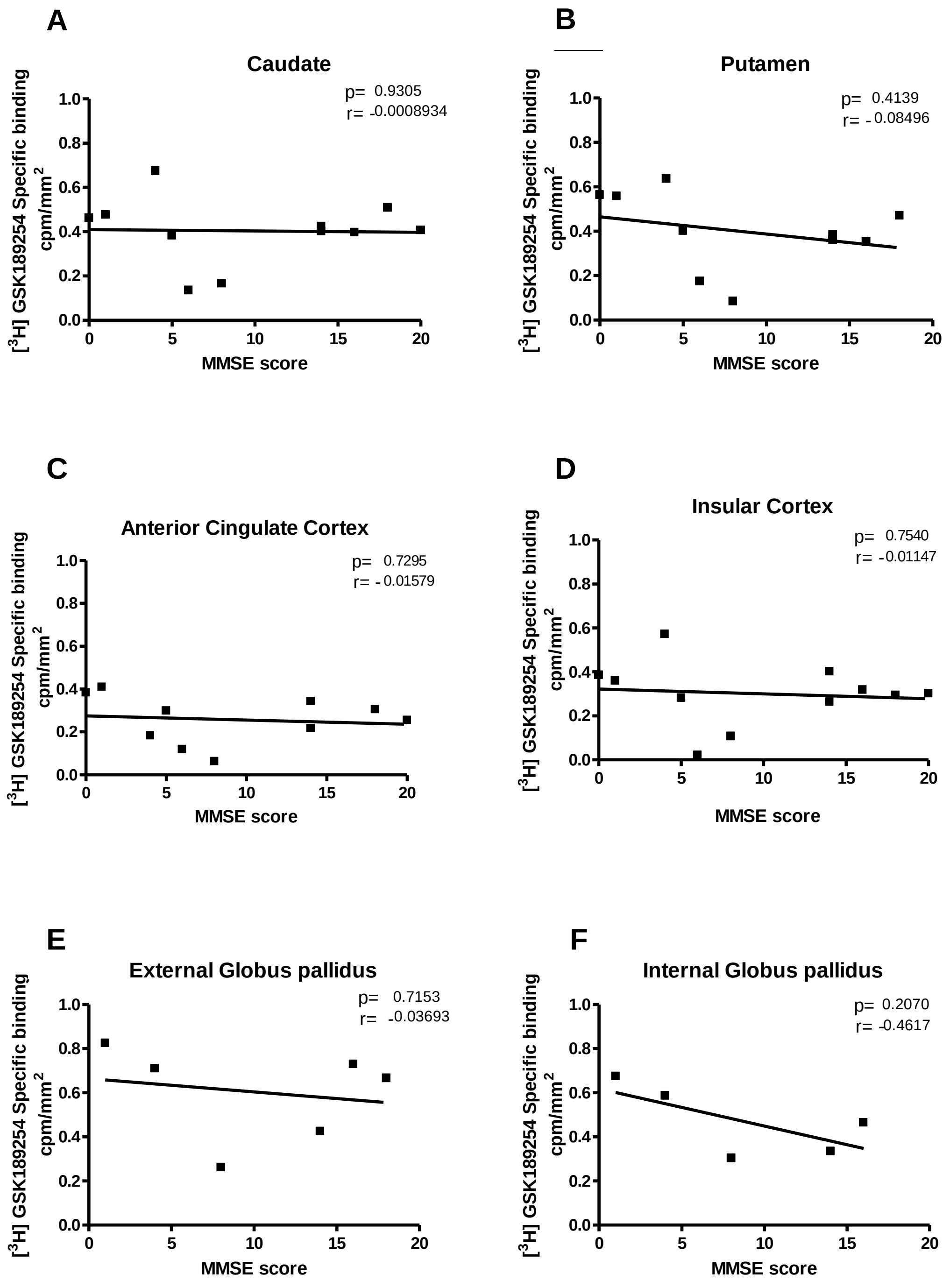

Supplementary Figure 2: Mini mental state examination score against specific binding cpm/mm^2 of [^{3}H] GSK189254 in AD cases in (A) Caudate, (B) Putamen, (C) Cingulate cortex, (D) Insular cortex, (E) external Globus Pallidus, (F) internal Globus Pallidus. No significant relationship was seen with the brain structures investigated. Each point is an individual patient case.

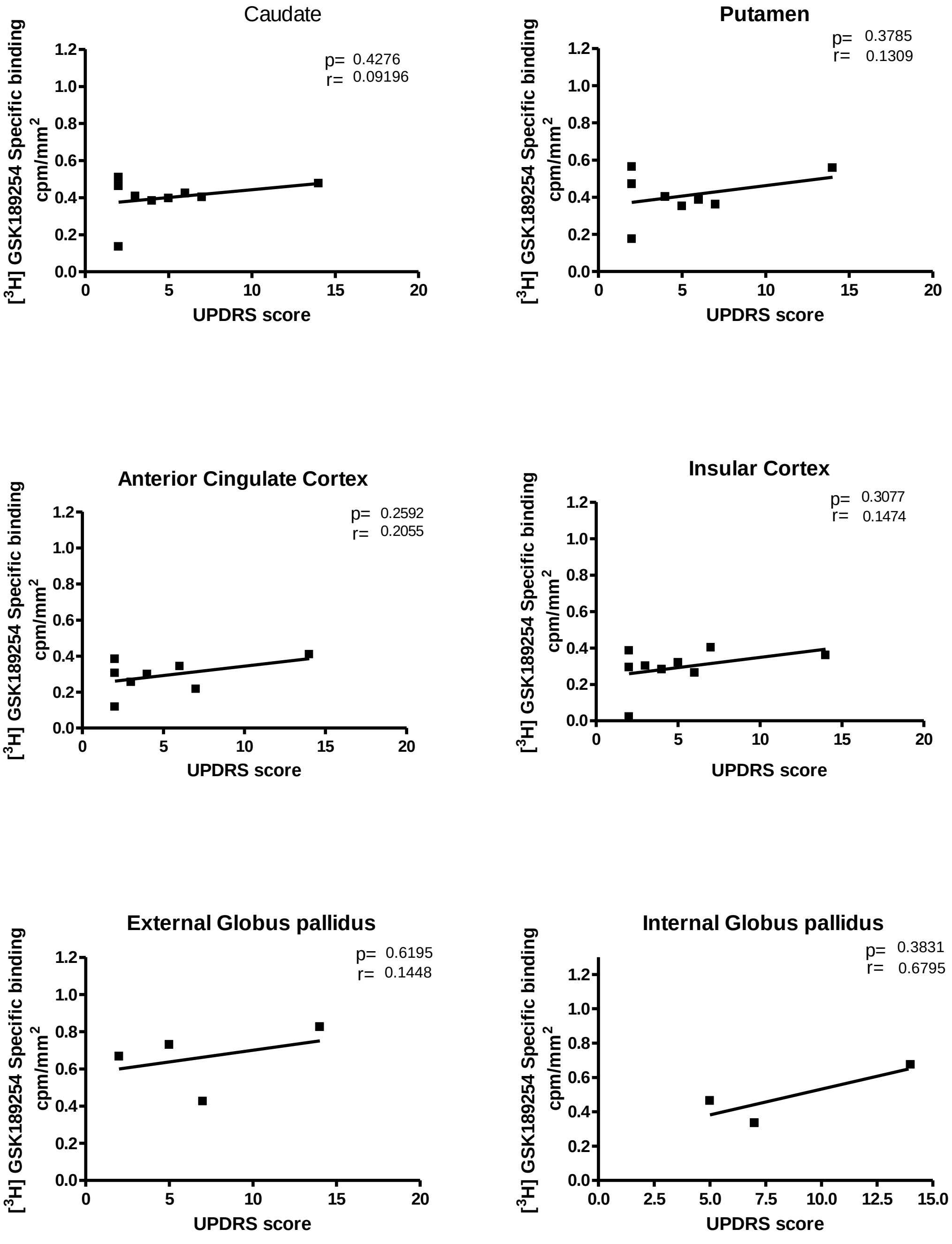

Supplementary Figure 3 Unified Parkinson Disease Rating Scale data against specific binding cpm/mm^2 of [^{3}H] GSK189254 in AD cases in (A) Caudate, (B) Putamen, (C) Cingulate cortex, (D) Insular cortex, (E) external Globus Pallidus, (F) internal Globus Pallidus. No significant relationship was see with the brain structures investigated. Each point is an individual patient case.

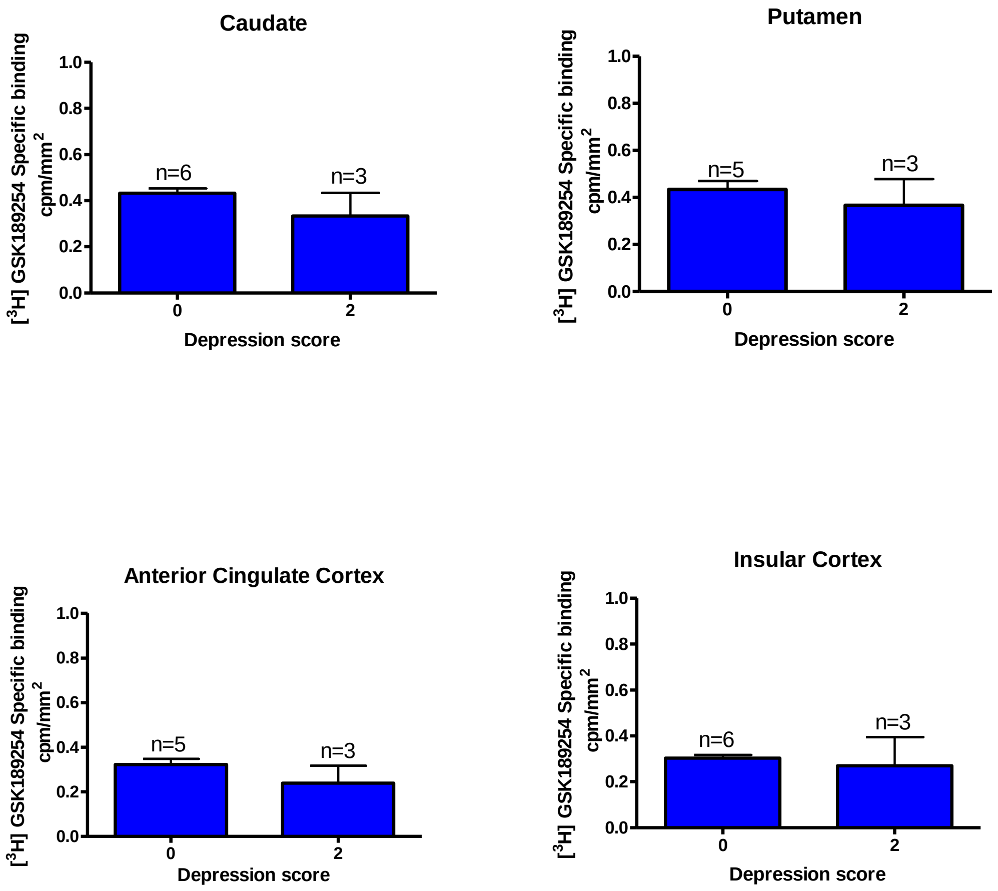

Supplementary Figure 4: Depression score against specific binding cpm/mm^2 of [^{3}H] GSK189254 for AD cases in (A) Caudate, (B) Putamen, (C) Cingulate cortex, (D) Insular cortex. No significant differences were observed.

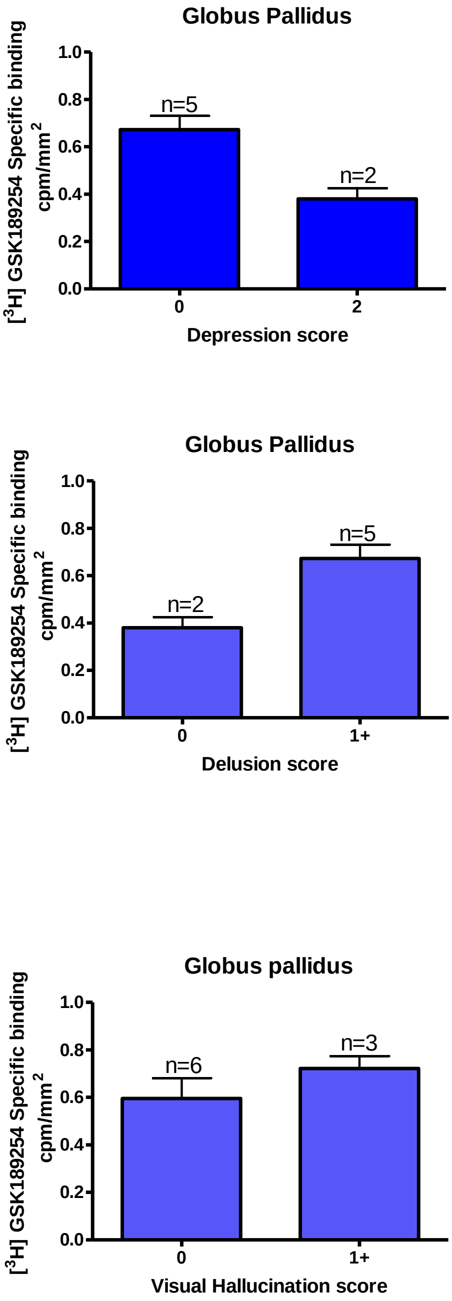

Supplementary Figure 5: Correlation of depression, delusions and Visual hallucination scores against specific binding [^{3}H] GSK189254 binding for AD cases in Globus Pallidus (n = 2-6 individual cases). There was a trend for elevated levels related to delusions, but not formally analysed due to small numbers of cases.
